# Twelve Letters, with Remarks

**Published:** 1873-04

**Authors:** 


### Our Correspondence.

### Letters Exposing Humbugs and Swindlers,

**With other Communications of Interest to the People.**

LOWELL, MASS., JANUARY 4, 1873.

*Dear Sir :*—Will you inform us through the BISTOURY, why Dr. Peaslee, in giving statistices of operations for ovariotomy, selects some to the neglect of others in the same region, that are well known to him ? If statistics have any value, it is in the truthfulness of them as given to the public. B.

The above is from a distinguished surgeon, who has operated one hundred and sixty-seven times oftener than Dr. Peaslee has, for ovariotomy. Dr. Peaslee recently published a work entitled "Ovarian Tumors; their Pathology, Diagnosis and Treatment, especially by Ovariotomy," in which he professes to give the number of ovariotomies, and the names of those who have performed a number of operations, yet he entirely ignores many surgeons who have operated many times more than he has. No mention is made of Dr. Walter Burnham of Lowell, Dr. Moore of Rochester, Dr. March of Albany, or ourself, all of whom have had as many successful cases, and Dr. Burnham a great many *more*, than Dr. Peaslee.

It perhaps can only be accounted for upon the theory that Dr. Peaslee is jealous of his honors, and is loath to mention names more prominent, as ovariotomists, than his own.—Twenty-eight operations is a very slender number from which to deduct conclusions covering 550 octavo pages. If this is the orthodox number entitling one to rush into print as an author, a deluge of books upon this subject is about to burst upon an unsuspecting community.

---

A STARTLING COMMUNICATION.

I never orded your Book and there four you Better stop it for I shant not pay one cent nor you Cat gitet and you Can drive your teem wethers filld——springs January the 30 th

JOSEPH PACKARD

No remarks !

---

LAWREN'S C. H., S. C., Feb. 13, 1873.

DR. UP DE GRAFF :

I have received four duns recently to pay up. I sent you $2, which pays up, and one year in advance. Some mistake, please rectify it. What would you do with a negro's heel-string, gnawed in two by rats ? D. L. A.

1st. Your account on our books is straight. Bills were sent through error of mailing clerk.

2nd. Kill the rats !

---

HERKIMER CO., N. Y., Jan. 10, 1873.

DIRECTOR ELMIRA CITY HOSPITAL:

*Gents :*—Can you inform me when the "drawing" in the Elmira Premium Land Sale, for the benefit of the Elmira City Hospital, will take place. I hold several tickets, and am anxious to know something about it.

Respectfully yours, G. D.

The above drawing will occur when innocents, like you, will cease patronizing lotteries, traveling doctors, patent medicines, and other swindles, viz : NEVER !

---

SHERMAN, TEXAS, FEB. 16th, 1873.

DR. UP DE GRAFF :

*Dear Sir:*—Enclosed find remittance to square our account.

Would be pleased to have the recipe for making a cure for itching in the head. You sent a recipe to me once, and it cured my constantly itching head. Did not suppose I would ever need it again, and gave it away.—If you can send it, you will much oblige.

J. W. H.

Have your druggist prepare the following :

Bay rum, 1 pint ;
Tincture of cantharides, 1 fluid ounce ;
Carbonate of potash,
Carbonate of ammonia, of each, 15 grs.

Dissolve the carbonates in a little water, then mix with the other liquids. Wet the scalp with the preparation, and brush it in thoroughly with a stiff brush, after which wash with warm water. Rub the scalp and hair dry, then use pure bay rum as a final dressing. Treat your scalp in this manner twice a week, when all itching and dandruff will disappear.

---

MOSCOW, PA., FEB. 14, 1873.

*Dear Doctor :*—I have been a sufferer for the past five or six years—cause pronounced liver complaint. Irregularity of the bowels the greatest trouble. Our physicians here, give me no permanent relief.

If I had not that Colt case in mind, I would ask you, if you could prescribe something in your next issue that might assist our M. D., in his efforts to do me some permanent good. If so, and I am benefitted thereby, I would ever be thankful that the Bistoury was addressed to me.

Respectfully and truly yours.

C. A. H

Your family physician is the best judge of what is needed in your case. It is impossible for us to prescribe for you with so slender an understanding of your case.

Prescriptions for constipation and "anti-billious" pills will be found in our Medical Items and News. Refer them to your physician who may approve or reject them in your case.

---

Harrisburg, Pa., March 1, 1873.

Dr. Up de Graff:

Do you know anything about Drs. Buchanan and Kline's "Great Cancer Specific?" These parties claim to be professors in a Medical College in Philadelphia, and offer to cure cancer in all its forms and stages. Would you trust them? A. R. M.

Scarcely! The cancer specific, college, (?) and doctors, are considered humbugs. Their college charter was cancelled by the Pennsylvania Legislature last winter, by reason of the concern, and those connected with it, being first class frauds.

---

Susquehanna, Pa., March, 20, 1873.

Editor Bistoury:

We have a little girl, eight years old, who is troubled with curvature of the spine. What can be done for her? P.

Take her to some skillful surgeon, who will prescribe a suitable brace or support for her. Do not delay, the sooner mechanical treatment is adopted, the better the prospects for cure.

---

Ithaca, N. Y., Feb. 28, 1873.

Dr. Up de Graff:

Please tell me in next issue of the Bistoury what to do for my little boy, 2 years old, who stands upon the outer side of his foot,—his foot turning in. Mrs. R.

Have a suitable club foot apparatus adjusted to the case, and perhaps the contracted tendons cut. A competent surgeon can only decide.

---

Penn Yan, N. Y., March 5, 1873.

Dr. Up De Graff:

I read Bistoury, find many replies to correspondents, so take the liberty to ask a question. My eyes are failing me. I cannot see plainly, they blur after reading awhile, and then dark floating spots appear before them. I am 38 years old, yet must wear glasses suited to a person of sixty or seventy years of age, before I can see reading matter plainly.—What do you suppose the trouble is? B.

The trouble is *not* in the refractive power of your eye, and, if you continue the use of glasses, the probabilities are that you will render yourself entirely blind. You are highly sensurable for employing glasses without the consent of an oculist. The difficulty, most likely, exists in the the retina, which may be congested, or otherwise diseased, and which can only be detected under an ophthalmoscope, in the hands of a competent oculist.

Discontinue the use of glasses at once, and seek the advice of some one versed in such matters, and do not again trust your eyes to an incompetent spectacle vender.

---

Harrisburg, Linn Co., Oregon, January 23, 1873.

The Bistoury:

Enclosed three dollars and fifty cents, for Peterson's Magazine and Bistoury to——, and Young Folks' Rural and Bistoury to——, to Harrisburg, Linn County, Oregon.

I enclose flowers picked from my garden this morning, on the way to my store. I could gather large boquets of many kinds of flowers, both in garden and all over the valley. I have not seen any snow or ice, and but little frost, this winter. Grass, green and growing.—Stock doing well on native grass. Farmers plowing, sowing grain, &c.; our valley is 40 to 50 miles wide, by 150 miles long. Our State has more square miles than your great State, and only 100,000 inhabitants. Our farmers raise from 30 to 40 bushels of wheat per acre. I enclose a few grains for your examination.—Land cheap, climate very healthy. I have lived here twenty years with my family. From the despatches, I see it's very cold, with snow, in Atlantic States. Why don't people emigrate to this country from the old States of the Union? I live in a latitude 45 degrees north of any part of your State. I own a thousand head of stock, have not fed them any this winter, and don't expect to. Have kept about that number for twenty years. Have not fed but two months during the whole time. If you can't answer in the BISTOURY, have a brother editor give reasons why people don't come to this country. We have railroads, churches and fine school houses all over this valley (Welliamett). When I came, there was nothing but nature, not a house, except board shanties, to live in. We have both timber, prairie, and level land. Winds blow from the north in summer, and south in winter. Rarely thunders. I have heard it thunder but twice since I've been here.

Yours, HIRAM SMITH, Postmaster.

N. B. Any number of bachelors here, with good houses, and no old maids.

Mr. Smith's question is a poser. Am sure we cannot tell *why* men of limited means, do not prefer a country of the character described by our correspondent, where land is cheap, and climate glorious, to remaining here, when the price of an acre costs as much as a large farm, in Oregon. A friend, to whom we have just submitted the matter, gives the following solution to the problem: Take Brown, as an illustration, who has earned or inherited a small farm, that affords him a comfortable living. He is surrounded by pleasant neighbors, good schools, fine churches,—with preaching talent superior to that found so far west; has a contented and loving wife, and bright and happy children. Now, *he* doesn't want to "go west," and why the d—l should he?

Then take Black; he's a loafer,—never earned enough to buy even a foot of western land, much less a farm; *he* would like to reside in the salubrious climate referred to, and own a farm, too, but he has not the means to defray the expenses of transportation, much less purchase lands. Of course, it is plain that *he* can't go west,—and why should he? As a middle class, we have Green; he has some means, is discontented, wants to get rich faster; sees Mr. Smith's letter in the BISTOURY, and concludes he'll migrate. He, with a few congenial spirits, locate in our correspondent's locality, cultivate the rich and productive soil, and soon raise more wheat and potatoes than can be disposed of at remunerative prices; in fact, "glut the market." It will then be our correspondent's turn to ask the question—why in thunder did they come here?

---

MASTER JAMES FINLAY MUST TRY AGAIN.

MT. MORRIS, March 1873.

*Editor Bistoury:*—In your last issue I notice an article under the caption "Spelling," in which "Master James Finlay carried off the prize, having spelled eighty-three words of the hundred correctly."

While we would say to Master James, well done, yet we feel proud to say that Miss Clara Ames (aged twelve years), of Union School No. 2, of our village, spelled ninety-five of the hundred words, of the same list, correctly. It is but justice to Miss Clara to say that she spelled off-hand, the words being pronounced to her by the writer. She says she could have done better if she had written them, as Master James did. W. R. WELLS.

Miss Clara can go up head.—Next!

